# Imagining the cell therapist: Future CAR T cell monitoring and intervention strategies to improve patient outcomes

**DOI:** 10.1002/jha2.357

**Published:** 2021-12-08

**Authors:** Michael D. Jain, Jay Y. Spiegel

**Affiliations:** ^1^ Department of Blood and Marrow Transplant and Cellular Immunotherapy Moffitt Cancer Center, and Department of Oncologic Sciences Morsani College of Medicine University of South Florida Tampa Florida USA; ^2^ Division of Transplant and Cellular Therapy Sylvester Comprehensive Cancer Center University of Miami Miami Florida USA

**Keywords:** cell therapy, lymphomas, myeloma

## Abstract

Chimeric antigen receptor (CAR) T cell therapy is now approved for the standard of care treatment of several types of relapsed or refractory hematologic malignancies. Future advances may extend cellular therapies to solid tumors or even non‐malignant diseases. As patient need grows, a clinical specialty of “cell therapy” may emerge. Here, we envision the needs of a clinical cell therapist to monitor and intervene upon patients receiving cell therapies. These include: (1) monitoring patient T cell quality and the host immune environment to ensure optimal timing for cell therapy. (2) Tumor antigen profiling to personalize CAR T cell targeting. (3) Real‐time monitoring of CAR T cells and circulating tumor DNA to modulate CAR T cell activity to maximize tumor eradication while mitigating toxicity. (4) Monitoring of CAR rejection and anti‐CAR immunity posttreatment to inform re‐dosing and subsequent cell therapy strategies. Armed with these tools, the future Cell Therapist may optimize and personalize treatment to avoid toxicity and improve efficacy universally across CAR designs.

## 1 INTRODUCTION

Clinical adoptive cell therapy, or the use of cells as drugs, is now a reality for many patients with hematologic malignancies. Chimeric antigen receptor (CAR) T cell therapy is currently approved as standard of care for the treatment of relapsed or refractory (R/R) large B cell lymphoma (LBCL), mantle cell lymphoma (MCL), follicular lymphoma (FL), B‐cell acute lymphoblastic leukemia (B‐ALL) in children and adults, and multiple myeloma (MM). However, a challenge of CAR T cell therapy is that treating physicians are unable to monitor the progress of the infused cells and do not have the tools to guide personalized interventions that could improve outcomes. Here, we consider and discuss the near‐future tools and interventions that a successful Cell Therapist could use to improve patient outcomes. While other reviews have considered the future of CAR T cell therapy from an engineering perspective (i.e., CAR design) [1, 2], this review focuses on the clinical needs of a cell therapist that could be used across CAR designs and cancers.

Currently, approved CAR T cell products are produced from the patient's own T cells (e.g., autologous T cells) after collection through a leukapheresis process. These cells are sent to a manufacturer's laboratory where they are transduced with a CAR and stimulated to promote CAR T cell proliferation and grow an adequate product. After 2‐3 weeks the CAR T cells are tested and, if meeting release specifications, are sent back to the treating hospital. Patients then receive conditioning chemotherapy followed by infusion of their CAR T cells. Standard CAR designs utilize a tumor‐recognition receptor, such as a fragment of an anti‐CD19 antibody (B‐cell malignancies) or anti‐BCMA (myeloma). Binding to the tumor triggers activation of the CAR T cell to proliferate, release cytokines, and differentiate into effector CAR T cells that eliminate the tumor cells. CAR T cells multiply inside the patient to reach a peak 7‐14 days later. During this time, patients may experience toxicities known as cytokine release syndrome (CRS) and immune effector cell associated neurotoxicity (ICANS). After reaching their peak, the CAR T cells rapidly contract and the side effects subside. Depending on the disease, up to 40‐50% of patients may attain durable remissions, but the remainder relapse. Unfortunately, outcomes are typically poor post‐CAR T cell therapy relapse [3, 4].

While the above is well recognized, clinicians are unable to dynamically monitor CAR T quality for individual patients. Few centers evaluate T cell subsets present at the time of patient leukapheresis, and manufacturers provide little information about the subsequent CAR T cell product other than the dose. Once CAR T cells are infused, clinicians are unable to easily monitor cytokines or CAR T cell levels, instead relying on crude inflammatory markers such as C‐reactive protein (CRP) and ferritin, along with clinical symptoms, to divine a patient's progress. Similarly, tumor responses are not checked until at least a month after infusion and by that time the CAR T cells have mostly disappeared and the window for productive intervention may have closed. Finally, there are limited clinical tools to follow anti‐CAR T immunity in survivors, and thereby identify which patients may benefit from further cell therapy versus other treatments.

**FIGURE 1:**
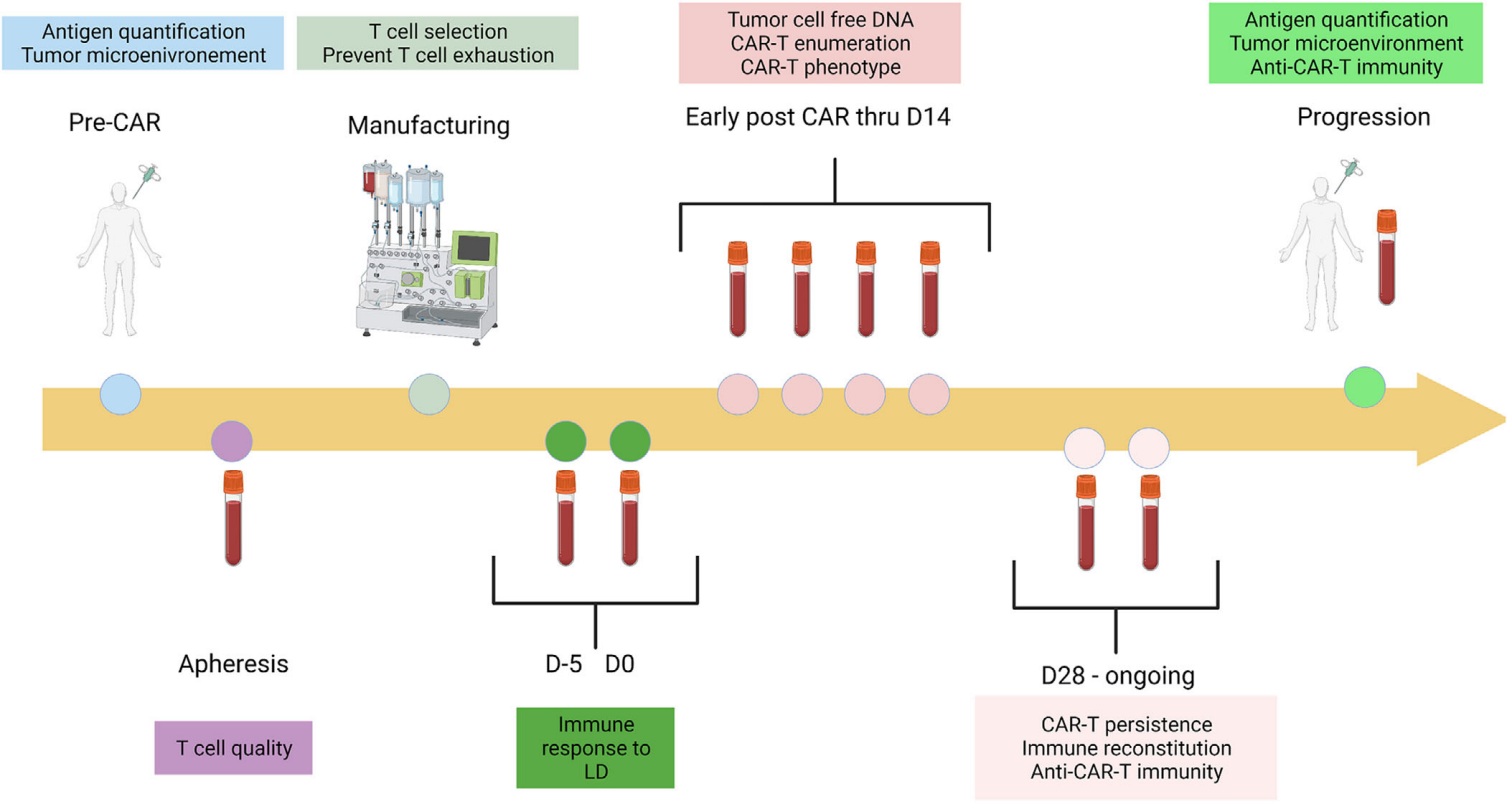
**Monitoring for the future cell therapist**. To comprehensively monitor patients proceeding through cell therapy, assessments will be required at multiple times. Prior to therapy, the physician will need to determine tumor characteristics such as antigen density and patient T cell phenotypes through the collection and manufacturing process. After infusion, tumor ctDNA will determine adequate treatment response and CAR T cell monitoring will ensure ideal expansion and maintaining of function. If relapse occurred, the patient could then be assessed for fitness for future cell or alternative therapies. Image created with ©BioRender ‐ biorender.com as licensed by the Scientific Development Office of the Moffitt Cancer Center

In this review, we will discuss tools that would be useful to a cell therapist to optimize and personalize clinical treatment (Figure 1; Table 1).

**TABLE 1:** Monitoring for the future cell therapist

**Category**	**Use**	**Example**	**Needs**
T cell Quality	Determine timing of leukapheresis for autologous CAR T	Can I prescribe a treatment to improve T cell quality? How long to washout chemotherapy before leukapheresis? Select optimal T cells from which to make CAR T product.	Define T cell quality
Host environment	Determine when to infuse CAR T cells	Is the patient optimally lymphodepleted? Can I prescribe a treatment to decrease systemic T cell suppression?	Define a favorable host environment
Tumor Antigen Profiling	Determine if target expression is sufficient for CAR success	Quantify tumor CD19 prior to anti‐CD19 CAR T cells	Define cut‐offs for efficacy across different CAR T cell products
Real‐Time CAR T cell monitoring	Follow CAR T cell levels and subsets after infusion.	Early toxicity management in patients with high expansion. Intermittent “rest” of CAR T cells that are becoming exhausted.	Develop interventions to allow CARs to be “driven” early after infusion.
Tumor response	Optimize CAR T cell dosing	Should patients with early positive ctDNA receive more CAR T cells?	Real‐time monitoring (i.e., ctDNA).
Anti‐CAR immunity	Determine risk of rejection to a given construct	Should a patient be re‐infused with the same CAR at time of relapse?	Easily scalable assays

## 2 MONITORING PATIENT T CELL QUALITY AND THE HOST ENVIRONMENT

### 2.1 Monitoring and modulating patient T cell quality

As CAR T cells are patient derived, the quality of patient T cells affects the quality of the manufactured CAR T cell product. However, there are currently no clinical markers of a patient's T cell quality that can guide a Cell Therapist when considering a patient for leukapheresis. One issue is that the definition of T cell quality in the context of CAR T cell therapy still needs refinement. Working towards this goal, recent studies have analyzed leukapheresis material in relationship relation to CAR T cell product and patient outcomes. For example, in the ZUMA‐1 clinical trial of axicabtagene ciloleucel (axi‐cel) in LBCL, a shorter T cell in vitro doubling time during manufacture was found to associate with a better outcome [5]. However, the T cell doubling time depended on the types of T cell subsets obtained from patient leukapheresis. In general, the more stem‐like memory T cells (and fewer effector T cells) in the leukapheresis material, the shorter the doubling time, and the better the CAR T cell product [5, 6]. Similarly, researchers at the University of Pennsylvania studied patients with pediatric B‐ALL, comparing leukapheresis material characteristics between patients who had CAR T cells with long (good) versus short (bad) persistence after infusion [7]. Again, better persisting CAR T cells originated from less differentiated T cells, while short persisting CAR T cells originated from leukapheresis enriched for effector T cells. Furthermore, T cells with high expression of gene targets of type 1 and 2 interferon (IFN) resulted in short persisting CAR T cells, highlighting that poor T cell quality is related to patients with chronic inflammation. Overall, the causes of poor T cell quality are likely related to tumor‐driven immunosuppression and inflammation, T cell injury due to chemotherapy, and patient‐specific factors such as age.

The problem of poor T cell quality may be partly solved by improved CAR T cell manufacturing. Here, manufacturing would select the most optimal T cell for CAR transduction and/or use in vitro methods to improve the quality of the obtained T cells. Preclinical work in the Riddell lab demonstrated that CAR T products of CD4^+^ naive T cells and CD8^+^ central memory T cells improved tumor eradication in a mouse model [8]. Operating from a similar principle, lisocabtagene ciloleucel, a CD19 CAR T cell therapy approved for LBCL, is infused in an equivalent ratio of CD4:CD8 CAR T cells [9]. Another strategy to improve manufacturing is to remove suppressive cells. Several studies have shown that suppressive myeloid cells, accumulated during the leukapheresis procedure, decrease the success of CAR T cell manufacturing [10, 11]. Other studies have suggested that these myeloid cells may even be transduced by CARs and, once infused, cause toxicity [12]. Alternatively, T cells can be cultured using various inhibitors (i.e., an inhibitor of PI3‐kinase, or tyrosine kinase inhibitors such as dasatinib), to result in more potent manufactured CAR T cells, potentially rescuing even poor quality T cells [13, 14].

An alternative solution to the problem of T cell quality may be to instead use allogeneic T cells from a healthy donor [15]. Donor T cells could be selected for high quality and result in manufactured CAR T cells with high polyfunctionality and a desirable T cell subset composition. However, allogeneic CAR T cells need to overcome the problem of “host versus graft rejection,” where the allogeneic CAR T cells are prematurely rejected by a patient's immune system. Strategies to overcome this problem include the use of more intensive lymphodepletion/immunosuppression, or cell engineering to hide the CAR T cells from host immunity. However, certain allogeneic CARs have led to CAR‐derived T cell lymphomas, leading to concerns about some types of cell engineering and the potential for oncogenesis [16, 17]. While the CAR transgene insertion into an oncogene has been observed in autologous CAR T cell therapy, clinical experience to date suggests that this problem (or the related problem of CAR insertion into an already malignant B cell) are comparatively rare events [18, 19].

### 2.2 Monitoring and modulating an unfavorable host environment

Despite tangible solutions to improve CAR T cell quality, there is still the problem whereby cancer patients have systemic immune dysfunction. Even the best CAR T cells may not work if infused into a host environment that immediately causes T cell dysfunction. Recently we showed that tumor burden and the tumor microenvironment (TME) affect systemic inflammation and CAR T cell expansion in LBCL [20]. We found that the expression of genes in the TME associated with chronic tumor IFN signaling is associated with poor CAR T cell expansion and a lack of durable responses. Interestingly, these same IFN signaling genes were found by the University of Pennsylvania group to associate with poor T cell quality and short CAR T cell persistence upon manufacturing and patient treatment [7]. Therefore, it may be necessary to treat inflammation or other pathways within the tumor to improve pre‐apheresis T cell quality and/or improve the host environment post‐infusion. A possible example of this is the treatment of chronic lymphocytic leukemia (CLL) with ibrutinib, a kinase inhibitor that targets BTK on the malignant B cells and ITK on the T cells [21, 22]. Patients with CLL have decreased levels of multiple T cell subsets and carry T cell functional deficits. Upon treatment with 5 or more months of ibrutinib, patients exhibited improved T cell numbers and function, and upon CAR T cell manufacturing exhibited improved CAR T cell functionality. The benefit of ibrutinib was due to not only its effect on T cells but also by decreasing CLL‐induced immunosuppression. Indeed, a clinical trial recently described the initiation of ibrutinib 2 weeks before leukapheresis followed by concurrent treatment with CAR T cell therapy for CLL. Compared to a cohort without concurrent ibrutinib, CAR T toxicity was less severe but similar long term outcomes were observed [23]. Ibrutinib may not be entirely successful in this regard, and new approaches are needed. Understanding and monitoring the impact of pre‐CAR treatment on T cells and the malignancy associated immune environment could help a cell therapist optimize the timing of leukapheresis and CAR T cell infusion.

Another strategy could be to improve pre‐CAR conditioning regimens. Lymphodepleting chemotherapy is typically given a few days before the CAR T cell infusion to limit host anti‐CAR T cell immunity and to establish a cytokine environment within the patient that is conducive to CAR T cell expansion. CAR T cell expansion is greatly affected by lymphodepletion intensity, and the success of lymphodepletion may depend on the induction of specific cytokines such as IL‐7 and IL‐15 [24, 25]. The current standard for lymphodepletion uses fludarabine and cyclophosphamide (Flu/Cy). However, we found that systemic inflammation and an unfavorable cytokine environment were not overcome by Flu/Cy in patients receiving CAR T cell therapy for LBCL [20]. We measured levels of ferritin, CRP, and inflammatory cytokines such as IL‐6 in patients immediately prior to lymphodepletion, after lymphodepletion on the day of CAR T cell infusion, and at peak in the first month. Overall, Flu/Cy lymphodepletion did not overcome the unfavorable inflammatory state, and these problems were amplified upon infusion of CAR T cells, leading to excess toxicity. We also found that peripheral blood myeloid‐derived suppressor cell (MDSC) levels are associated with poorer CAR T cell expansion [20]. Therefore, targeting MDSCs before CAR T cell infusion may be of benefit. Successful conditioning would lead to patients having low levels of MDSCs and inflammatory cytokines such as IL‐6, but higher levels of cytokines associated with benefit, such as IL‐7 and IL‐15. The optimal conditioning regimen may differ depending on the nature of the immunosuppression or inflammation present in an individual patient.

In an ideal future, a Cell Therapist would be able to monitor patients’ T cell quality and host environment. Clinicians currently do not know the effect that our treatments have on these parameters. How long should we wash out certain treatments before apheresis to allow recovery of T cell quality? How long do we need to treat patients with, for example, ibrutinib, to improve T cell quality? Can measurement of T cell quality predict in advance which patients are likely to fail autologous CAR T cell manufacturing, since this is often catastrophic for patients? Are there any interventions to improve the host environment before CAR T cell infusion? When giving therapy between leukapheresis and the start of conditioning (“bridging”), should we give chemotherapy, radiation, corticosteroids, or specific novel agents? Should we modify or intensify conditioning in some patients? The answers to these questions may be universal, but they may also be unique to individual patients, different for different cancers, or may be specific to certain CARs and manufacturing processes. In the future, individualized monitoring of T cell quality and host environment could help cell therapists optimize patient treatment.

## 3 TUMOR ANTIGEN PROFILING

At present, approved CAR T cell products target a single antigen, either CD19 in B‐cell lymphomas and leukemias, or BCMA in myeloma. One mechanism of resistance to monospecific CAR therapy is inadequate target antigen for CAR T cells prior to infusion or loss/decreased antigen expression after CAR T [26, 27]. In the clinical trials leading to approval of these agents, expression of antigen prior to therapy was not found to impact response to therapy. However, in the case of lymphoma and myeloma, targets (CD19 or BCMA) were largely measured by immunohistochemistry. In patients treated with axi‐cel for LBCL, we recently demonstrated that quantitative flow cytometry can find differences in CD19 molecules/cell despite high CD19 expression by IHC [26]. These differences were important, as those with lower levels of CD19 were more likely to experience disease progression. For the future cell therapist, methods to profile antigen expression levels may be useful to guide treatment. For example, preclinical data has shown differences between approved constructs based on the costimulatory molecule employed, as CD28 co‐stimulated CAR T cells are better able to clear lower levels of antigen relative to CARs stimulated by 4‐1BB [28]. In LBCL, some of the approved products are co‐stimulated by CD28 (axi‐cel) while others are co‐stimulated by 4‐1BB (tisa‐cel and liso‐cel), and antigen levels could be used to guide product selection. This approach may be of specific importance to patients previously treated with non‐CAR therapies against the same or similar targets. For example, patients with B‐ALL may be treated with the CD19‐targeted bispecific T cell engager blinatumomab. In a recent abstract, patients who received prior blinatumomab were more likely to have dim CD19 on their leukemia cells by flow cytometry prior to CD19 CAR T cell therapy and were more likely to experience treatment failure [29], although this has not been observed with all CAR designs [30]. Similarly, patients with R/R LBCL are now approved to receive the CD19‐targeting agents loncastuximab teserine or tafasitimab and R/R myeloma patients may receive the BCMA‐targeting drug belantamab [31, 32, 33]. Whether these therapies require different antigen expression levels to be successful, and how often relapse after these therapies results in decreased antigen levels remains to be determined. Better measures of the target antigen prior to therapy could distinguish patients at risk for treatment failure, particularly when patients have received an agent targeting the same antigen as the CAR. Similarly, even in the setting of adequate target prior to CAR T cell therapy, antigen loss, or decreased expression may occur at relapse. In B‐ALL, the rate of CD19 loss at the time of relapse is approximately 50% across pediatric and adult clinical trials [34]. Intron retention and splice variants have been described as etiologies of CD19 loss and lineage switch to AML has been described in patients with KMT2A rearranged ALL [35, 36]. The rate of CD19 loss in large cell lymphoma is less than B‐ALL but still ∼30% [3, 27]. With shorter follow‐up, the rate of BCMA loss appears less, although bi‐allelic loss of BCMA has been described and lower BCMA expression post infusion has been observed in up to two‐thirds of patients (with BCMA decreasing also in responders) [37, 38]. Currently, there is no standardized clinical test to assess tumor antigen levels before or after CAR T cell or other targeted therapies.

Potential solutions to the problem of low antigen expression prior to therapy could be addressed through multi‐antigen targeting, targeting a different antigen, use of a receptor that is effective at lower antigen levels, or methods to increase target expression prior to therapy. CARs targeting multiple antigens, both dual and triple CARs, are being tested in the clinic with varying success [26, 39, 40]. Additionally, monospecific CARs targeting other B‐cell antigens such as CD22 and CD37 are also in clinical trials [41, 42]. Knowledge of the antigen density of these targets could lead to rational selection of the best CAR T cell construct for each patient. Agents that serve to increase antigen density could also be employed in specific patients. For example, bryostatin has been shown to increase CD22 on tumor cells and has demonstrated safety in clinical trials and could therefore be given prior to CAR for patients with low CD22 expression to try to improve CAR efficacy [43, 44]. Similarly, gamma‐secretase inhibitors increase BCMA levels on myeloma cells and are being studied in conjunction with CAR T cell therapy [45].

## 4 REAL‐TIME MONITORING OF CAR T CELLS AND TUMOR RESPONSE

It may be possible for a Cell Therapist to “drive” CAR T cells with increased precision to increase or decrease activity in response to a patient's clinical condition. An emerging principle of clinical CAR T cell therapy is the effector‐to‐target (E:T) ratio. In vitro, this refers to the ratio of CAR T cells to tumor cells added in an experiment, with higher E:T ratios resulting in better cytotoxicity. In patients, the ZUMA‐1 clinical trial of CAR T cell therapy in LBCL found that E:T predicted outcome better than tumor burden alone [5]. In these patients, the effectors were measured by peak CAR T cell expansion, while tumor burden was measured by CT imaging. CAR T cell expansion appears to increase with higher tumor burden until reaching patients with the highest tumor burden, whereupon expansion falls [5, 30]. Patients who have enough CAR T cell expansion to overcome their tumor burden obtain durable responses, while patients with lower E:T ratios do not. Ideally, a cell therapist would be able to match CAR T cell expansion to tumor burden. Excessive expansion is likely to result in excess toxicity, while insufficient expansion would result in cancer relapse.

The first step toward this goal is to track CAR T cell levels within an individual patient as they proceed through treatment. To date, most measurements of CAR T cell levels are obtained by PCR and are not reported in real time to inform clinical management. The onset of CAR T cell toxicity (CRS and ICANS) usually occurs within the first week after infusion and one could envision that the knowledge of CAR T cell expansion during this period would be a valuable adjunct to guide toxicity prevention. The simple use of the absolute lymphocyte count has recently been reported to associate with CAR‐T outcomes and can be readily measured daily, however, this measure does not measure the cell of interest and would be difficult to utilize prior to the development of toxicity due to the impact of lymphodepletion chemotherapy [46]. Flow cytometry may be more amenable to clinical monitoring. Antibodies targeting the CAR protein itself (anti‐idiotype) identify CAR T cells in patient's blood in the research setting [47], and lisocabtagene maraleucel contains an EGFR tag that allows for monitoring of CAR expansion [48]. Beyond determining the number of CAR T cells alone, flow cytometry may also report on CAR T cell phenotypes. After infusion CAR T cells typically decrease their clonal diversity and after initial activation and differentiation they transition toward decreased proliferative capacity and expression of immune checkpoint ligands [12, 49, 50]. It may be possible to intervene during this process and prolong proliferation and decrease exhaustion in some patients. For example, recent preclinical data have shown that exhaustion in CAR T cells may occur predominantly at the epigenetic level and small molecules such as dasatinib may reverse the exhaustion program [14]. Giving dasatinib pulses, essentially turning the CAR on and off, was shown in mouse models to have the best antitumor efficacy with less differentiated CAR T cells. This approach could therefore provide a cell therapist the tools to “drive” CAR T cell expansion, persistence, and avoidance of exhaustion, but requires the ability to detect and comprehensively phenotype CAR T cells in real‐time.

To optimize the E:T ratio within patients also requires real‐time measurement of tumor response to therapy. It is now recognized that the peripheral blood of cancer patients contains circulating tumor DNA (ctDNA) that may provide information about tumor biology and treatment response. In LBCL, ctDNA may be used to monitor response to CAR T cell therapy, with day 28 ctDNA levels out‐performing PET imaging for prediction of subsequent relapse [51]. In another study, ctDNA measured at day 7 after infusion distinguished between patients who would remain in durable remission versus those who would go on to subsequent relapse [12]. Therefore, ctDNA, which may be measured in the peripheral blood in conjunction with CAR T cell dynamics by PCR, could be used to monitor patients during therapy.

In an ideal future, tumor response would be monitored early after CAR T cell therapy. If insufficient tumor clearance occurred, antitumor efforts could be intensified. For example, if day 7 ctDNA levels associate with poor outcome, such patients could be selected for dosing of additional CAR T cells. Similarly, a CAR product with a different target, or even a short‐lived allogeneic CAR could be additionally dosed at that moment without requiring repeat lymphodepletion. While the toxicity profile of additional doses may be unwarranted for most patients, it may be an acceptable trade‐off for high risk patients who are not having efficient tumor clearance after the first dose. Conversely, if one were able to match CAR T dose to tumor response, the first dose could be lowered such that many patients with rapid tumor clearance would not require subsequent dosing and possibly experience less toxicity. A particularly good use for this strategy could be in adult B‐ALL. The June group recently reported on a clinical trial that initially observed excess toxicity after a single high CAR T cell dose and insufficient efficacy at a lower dose. Therefore, an amendment was made to fractionate the dose such that 10% was given on day 1, 30% on day 2, and 60% on day 3. Patients who experienced early CRS did not receive subsequent doses, such that only 7 out of 20 patients received all 3 doses in the trial. Overall, patients treated with the fractionated strategy had a much better toxicity profile and improved efficacy outcomes [52]. This type of study highlights that fixed CAR T cell dosing is not optimal for many patients. Indeed, a dose of CAR T cells represents a variable mix of T cell subsets and proliferative capacity, with wide variation in antitumor effects between patients receiving the same dose. Matching CAR T cell dosing in real‐time to tumor burden could optimize the E:T ratio within patients and improve overall outcomes. To allow cell therapists to intervene, real‐time technologies are needed to monitor CAR T cell expansion and tumor response as clinical E:T parameters.

### 4.1 Monitoring of CAR rejection and anti‐CAR immunity post‐treatment

The above sections provide the cell therapist with tools to improve the likelihood of durable response to a single CAR‐T infusion. However, most patients still relapse, raising consideration of additional CAR T cell therapy, whether re‐infusion of the same or a different product. Moreover, clinical CAR T cell therapy is currently limited to a single planned infusion, whereas better tumor control might be realized by multiple cycles. At present, all approved products, both anti‐CD19 and anti‐BCMA, are derived from murine monoclonal antibodies. These murine sequences are theoretically susceptible to both B and T cell‐mediated rejection of the CAR T cells. Rejection could be responsible for loss of CAR persistence and would limit the success of repeat CAR infusions. Several trials have demonstrated cellular immune responses to murine‐based CARs. Turtle et al. found patients with these responses demonstrated poor CAR expansion with a second infusion in an early series with small numbers of patients [53]. In a larger trial, the same group reported repeat infusion of the same CAR T cell product across B‐cell malignancies and found a relatively low CR rate of ∼20% [54]. Factors associated with improved progression‐free survival were addition of fludarabine to the second lymphodepletion regimen and use of a higher dose of CAR. Currently, testing for anti‐CAR immune responses is not commercially available and assays for T cell mediated responses are particularly demanding. For example, the ELISPOT (enzyme‐linked immune absorbent spot) is used widely to test for T cell responses. To test CAR immunogenicity, overlapping peptide pools covering the length of the CAR protein are generated. Subsequently, patient peripheral blood mononuclear cells are co‐incubated with these peptides. If there is a T cell immune response to the peptides, the T cells will produce cytokines such as IFN‐γ that are captured by antibodies coated to the surface of wells used in the experiment. Widespread use of this and other assays at the point of care may not be easily realized, and simplified assays are needed. However, positive anti‐CAR immunity could lead a cell therapist to avoid CAR re‐infusion and seek an alternate approach, such as a humanized or allogeneic CARs, or non‐CAR therapies. A recent trial in pediatric B‐ALL reported the use of a humanized anti‐CD19 CAR that was able to induce responses in patients previously exposed to tisagenlecleucel [55]. Clinical trials with humanized CARs have also been reported in lymphoma, with similar response rates and lower detection of anti‐CAR cellular responses [56]. As previously discussed, rejection of allogeneic CARs is presently one of the main hurdles to success and methods to address this include multiplexed gene editing and intensive immunosuppression. However, the more immunosuppression that is required, the less feasible it is to consider multiple doses. Finally, anti‐CAR immunity may be dynamic, waning over time, and susceptible to intervention. Quantifying and modulating the extent of anti‐CAR immunity would allow the cell therapist to recommend subsequent doses of the same or different cellular therapies at the time of relapse.

## 5 CONCLUSION

Adoptive T cell therapy in the form of CAR T cell therapy is now standard of care for the treatment of several different types of relapsed or refractory hematologic malignancies. As the number of patients receiving cellular therapies increases, specialized clinical knowledge is required. However, clinicians providing cellular therapies currently lack the tools required to provide optimal and personalized care to patients. We have identified several areas where better monitoring could help a Cell Therapist optimize care. These include (1) monitoring patient T cell quality and the host immune environment to ensure optimal timing for cell therapy. (2) Tumor profiling to personalize CAR T cell targeting. (3) Real‐time monitoring of CAR T cells and circulating tumor DNA to match CAR T cell activity to tumor eradication. (4) Monitoring of CAR rejection and anti‐CAR immunity post‐treatment to inform re‐dosing and subsequent cell therapy strategies. The availability of clinical monitoring in these areas could guide a Cell Therapist in the future treatment of patients across a wide spectrum of diseases and cell therapy products.
